# Perceptions of female teenagers in the Tshwane District on the use of contraceptives in South Africa

**DOI:** 10.4102/curationis.v38i2.1528

**Published:** 2015-10-22

**Authors:** Ntswaleng S. Tabane, Mmapheko D. Peu

**Affiliations:** 1 Ga-Rankuwa Nursing College, Ga-Rankuwa, South Africa; 2 Department of Nursing Science, University of Pretoria, South Africa

## Abstract

**Background:**

Perceptions of female teenagers in the Tshwane District contribute to the non-use and or discontinued use of contraceptives as evidenced by increased levels of unplanned pregnancies.

**Objective:**

The objective of this study was to explore and describe the perceptions of female teenagers in the Tshwane District on the use of contraceptives.

**Methods:**

A qualitative, explorative, descriptive approach was followed in this study. The population comprised of pregnant female teenagers who were purposively selected. Data were collected using unstructured individual interviews on a face-to-face encounter in a natural setting. Data were analysed using the discourse method of data analysis.

**Results:**

The following perceptions on the use of contraceptives emerged: Perceptions on the use of contraceptives, emotions, contraceptive effects, social pressure and education on contraceptives. Teenagers’ perceptions were predominantly negative with unfounded fears. Though the teenagers were aware of the importance of the use of contraceptives, motivation to pursue contraception was lacking. Teenagers verbalised to be uncommitted as well.

**Conclusion:**

Various perceptions of female teenagers in the Tshwane District on the use of contraceptives were explored and described. It was noted that all the teenagers interviewed had great remorse and feelings of guilt regarding their behaviour of not using contraceptives. Their need for re-education was cited and seen as motivational enough to encourage the use of contraceptives at primary health care settings. Therefore, the study recommended that health education programmes should be restructured to effectively influence the female teenagers’ perceptions positively and to promote the use of contraceptives.

## Introduction

A decade ago, the idea of contraceptive use as a means of birth control was the right of every woman at child bearing age. The rationale was to help reduce the escalating levels of unintended teenage pregnancies (Onyensoh & Tumba 2011:1; Ramathuba, Khoza & Netshikweta 2012:1). However, female teenagers appear to perceive contraceptive use differently (Appiah-Agyekum & Kayi 2013:43; Hagan & Buxton 2012:175). Female teenagers, who should delay early sexual activity to prevent unintended pregnancies, experience major conflicting ideas regarding identity, education, employment, use and non-use of contraceptives (Ehlers 2010:15; Kanku & Mash 2010:563; Mosha, Rueben & Kakoko 2013:1; Wang, Jian & Yang 2011:1). According to Najafi, Rahman and Juni (2011:51), the rationale for a large number of unintended pregnancies show low contraceptive use. The teenagers’ future plans are mostly thwarted by unintentional pregnancies whilst they are not yet ready to have children. These make an overwhelming impact on increased teenage pregnancy rates in the world at large (Ehlers 2010:15; Okanlawon, Reeves & Agbaje 2010:23).

## Problem statement

Contraceptive methods are available for free in the Tshwane District clinics for the purpose of controlling and preventing unplanned and unwanted pregnancies. Despite the freely available contraceptives, female teenagers between the ages of 15 to 19 years still conceive unintentionally. To date, literature reports that 63% of sexually active unmarried women of 15 to 19 years are not using contraceptives (Nalwadda *et al.* 2010:13). Whilst accompanying learners in the selected clinic, the researcher discovered, from unpublished statistics on record, that 11 to 28 teenagers aged between 15 and 19 years were assessed and diagnosed in one month with unintended and unwanted pregnancies. The researcher observed that the number of teenagers from the same age group that had Termination of Pregnancy (TOP) performed ranged between 11 and 24 in a month. These might be related to the low use of contraceptives, hence the researcher's concern and interest in the perceptions of female teenagers in the Tshwane District on the use of contraceptives.

## Background

Family planning allows people to attain their desired number of children, delay pregnancy and determine the spacing of pregnancies (World Health Organisation 2012:1). This is achieved through the use of various contraceptive methods such as traditional methods, lactational amenorrhoea method and morden contraceptives. Seutlwadi, Peltzer and Mchunu (2012:43) reported that, in South Africa, unintended pregnancies remained a public health problem. Unwanted pregnancies are often the result of not using contraceptives (Unwanted Pregnancies Rife: Contraception 2008:1). These contraceptive methods are provided in the form of injectables, tablets, abstinence, lactational amenorrhea and artificial devices such as the Intrauterine Contraceptive Device (IUCD), to name but a few. Contraceptives are made available at various service points where services are equitably distributed and made available to all who need them (South Africa, Department of Health 2003:19). Policies relating to the access to contraceptives were put in place to allow children aged 12 years and above to be provided with contraceptives on request (University of Cape Town Children’ s Institute 2010:17). However, the use of contraceptives remains low. To achieve the 2020 objective of reducing teenage pregnancy by 10% requires a high regard for contraceptive use as the key driver towards changing teenagers’ pregnancy rates (Tyler *et al.* 2012:3).

Contraceptive methods were universally introduced in the 1960's as a strategy to prevent and reduce unintended and unwanted pregnancies amongst child-bearing women (Bruckner, Martin & Bearman 2004:248; Wang *et al.* 2011:1). The contraceptives were available for free to facilitate accessibility, affordability and to enhance avoidance of unplanned pregnancies. Nalwadda *et al.* (2010:13) asserted that the knowledge of contraception was universally spread to promote the health of child-bearing women. Yet the use of contraceptives was largely inconsistent or not initiated at all. However, studies conducted on the perspectives on teenage pregnancy in Dundee revealed that some young females use contraceptives (Petcu 2011:4). The young women who were not willing to use contraceptives were at risk of unplanned pregnancies, which constitutes 46% of all pregnancies in Uganda between the ages of 15 to 24 years (Nalwadda *et al.* 2010:13). In a study on factors influencing contraceptive use and unplanned pregnancy, it became clear that a total of 616 out of 1018 women discontinued the use of contraception (Bafana 2010:25). In addition, Association of Reproductive Health Professionals (2008:1), Najafi-Sharjabad *et al.* (2013:181) and Ahanoun (2014:36) stated that teenagers’ personal factors, such as low perception of risk for pregnancy, lack of thought, the thought of promoting sexual promiscuity and carelessness contributed to inconsistent and lack of use of contraceptives.

South Africa established family planning programmes in 1974 (South Africa, Department of Health 2003:6). The services were appropriated throughout the country to improve women's health through birth spacing. The services were integrated with other services to improve access. Access to contraceptive services that facilitate utilisation by all women of reproductive age has been increased (South Africa, Department of Health 2012:26). According to the National Contraception and Fertility Planning Policy and Service Delivery Guidelines (South Africa, Department of Health 2012:33–38), contraceptive services are provided for free at different levels of care to augment coverage by the public sector. However, the use of contraceptives was found to be low with an estimated rate of about 25% leading to a high rate of 61% of unintended pregnancy amongst child-bearing women in Gauteng and the Northern Province (Bafana 2010:13; Wood, Maepa & Jewkes 2009:17). It is assumed that contraceptive use is perceived differently by various age groups. This served as motivation enough for the researcher to look into the perceptions of the teenage group involved in this study.

Aryeetey, Kotoh and Hindin (2010:26), National Contraception and Fertility Planning Policy and Service Delivery Guidelines (South Africa, Department of Health 2012:26–38) and *Children's Act Guidelines* (University of Cape Town Children’ s Institute 2010:17) reported that efforts were made worldwide to encourage use of contraception. However, in spite of all efforts, literature reports on contraceptive use by women suggest either lack of use of contraceptives, limited use and or inconsistency in usage (Okonlawon *et al.* 2010:19). Hagan and Buxton (2012:175) reported 82.2% of lack of use of contraceptives amongst Ghanaian adolescents aged 15 to 19 years. The underutilisation of contraception amongst teenagers motivated the researcher to conduct a study in order to explore the perceptions of female teenagers in the Tshwane District on the use of contraceptives.

### Aim of the study

The aim of this research was to investigate the perceptions of female teenagers in the Tshwane District on the use of contraceptives in a selected clinic, with the purpose of improving contraceptive use.

### Objective of the study

The objective of the study was to explore and describe the perceptions of female teenagers in the Tshwane District on the use of contraceptives.

## Research method and design

A qualitative, exploratory and descriptive approach was followed. Qualitative research is an emerging methodological paradigm that attempts to collect rich descriptive data in respect of a particular phenomenon or context with the intention of developing an understanding of what is being studied (Maree 2010:49–50). De Vos *et al.* (2011:65) indicated that a qualitative research paradigm was used to produce descriptive data in the participant's own written or spoken words and to elicit the participant's account of their perceptions. The researcher described and explored the perceptions of female teenagers in the Tshwane District on the use of contraceptives to gain insight and to develop a rich understanding of their perceptions. The design assisted the researcher to obtain comprehensive in-depth descriptive information.

### Population and sampling

The target population invited to participate comprised pregnant female teenagers aged 15 to 19 years attending ante-natal clinic (ANC), Post-Natal Clinic (PNC) and those who delivered babies in the Maternity Obstetric Unit (MOU) in the Tshwane District area. Non-probability purposive sampling was used to select participants meeting the eligibility criteria. A sample size of ten participants was sufficient to provide data saturation (Polit & Beck 2012:275). However, in addition to the proposed number, the researcher added five more participants to ensure the study's credibility.

### Context of the study

The study was conducted in a natural setting. It was in the North-West area of the Tshwane District in a selected clinic. This natural setting was used as it is the most preferred clinic, because it is the only clinic in the area providing MOU and TOP services, and the catchment area is not restrictive. A private room, which was within the premises of a selected clinic and away from other consulting rooms, was utilised.

### Data collection method

Data were derived from the participants through the use of unstructured face-to-face interviews. The purpose of unstructured interviews was to elicit information that enabled the researcher to achieve an in-depth understanding of the participant's perception and to elicit authentic accounts from the participants. The unstructured interviews were pre-tested prior to the collection of data, and this assisted the researcher in refining the research question. The unstructured individual interviews, which were recorded on a voice recorder, were conducted during a face-to-face encounter, after permission was obtained from the participants. The researcher used the recorder to capture the fuller record of the conversation (De Vos *et al.* 2011:359). She posed the research question: ‘what are the perceptions of female teenagers on the use of contraceptives?’, followed by probing, clarification, paraphrasing and reflecting to facilitate smooth communication, increase detailed exploration and ascertain clear understanding of what was communicated by the participants. The individual interviews were conducted for 30 to 45 minutes until data saturation was achieved.

### Data analysis

Data were processed and analysed using the discourse method of data analysis (Terre Blanche, Durrheim & Painter 2010:328) to organise and synthesise the research data. The researcher undertook preliminary data analysis initially during data collection. The full conversation the researcher conducted with the participants was transcribed verbatim. The coding process included repeatedly reading through the data to understand the inherent sense. Thereafter, the principal researcher analysed and examined the interview transcript in detail to identify the recurrent patterns of data to be considered for themes. Different sections of the data considered relevant for themes were labelled. Similar patterns of talk in the form of sentences, phrases and even paragraphs were elaborated upon, summarised and condensed into subcategories, which were eventually classified into thematic categories. The interview transcript was submitted to the expert researcher for refinement of the categories. The participants’ accounts of the phenomenon understudy were put together as five main categories with relevant subcategories under each.

### Demographics of the participants

Demographic data were collected to contextualise the findings from the female teenagers’ background. Participants interviewed ranged between the ages of 15 to 19 years. The majority of the participants were still at high school level, except for one who was at tertiary level. Out of a sample of 15 participants, only one was married and three claimed to have planned the pregnancies. Almost all participants were still under parental care; an exception was one participant who indicated that she was to be married. These participants experienced the pregnancies and births for the first time.

## Results

The participants’ accounts of the phenomenon under study were put together using five thematic categories as main headings with subcategories or themes under each.

Table 1 below reflects the perceptions of female teenagers in the Tshwane District on the use of contraceptives.

**TABLE 1:** Categories and subcategories on the perceptions of female teenagers on the use of contraceptives.

Category	Sub-category
Perceptions on the use of contraceptives	Positive perceptions
	Negative perceptions
	Ignorance
	Blaming
Contraceptive effects on teenagers	Side effects and myths
Social pressure	Peer influence
	Influence by significant others
Education about contraceptives	Need for more information
	Reinforcement of what is known

### Perceptions on the use of contraceptives

The study revealed the perceptions female teenagers in the Tshwane District had on the use of contraceptives. The perceptions were classified into four subcategories namely: positive perception, negative perceptions, ignorance and blaming.

A positive perception of the use of contraceptives was expressed by some of the female teenagers. Contraceptives, which are used to prevent women from becoming pregnant, were perceived as a good practice which was supported by the participants, who said:

‘Ok first thing is that those conca … the pill is for preventing pregnancy.’ (Participant 12)

‘To … ok to stop the child from appearing and then sometimes and the diseases.’ (Participant 7)

On the contrary, some of the female teenagers perceived contraceptive use negatively. Reluctance to use contraceptives was evidenced in the following statements:

‘Some teenagers do not want to use contraceptives because of maybe they think that either they will make you fat, change the way you look.’ (Participant 4)

‘Many students do not want to use contraceptives just because they see contraceptives as … You see contraception as nothing; you ask yourself why do I use it?’ (Participant 8)

Therefore, the participants refrained from engaging in this practice, thereby revealing their ignorance on contraception use:

‘I think us teenagers, we are ignorant of them. Uh! Ja, ignorant towards them. The fact that we know about them and we are told a lot about them at school, the clinic and in the communities, still we do not go do, practice them. We got taught also at home.’ (Participant 6)

‘I never thought I could become pregnant so I did not see the use of it, of prevention.’ (Participant 9)

The teachings done were ignored such that perceived blaming of nurses’ behaviour was cited as reason for failure to use contraceptives and this became a strong hold which was described by female teenagers as: Prior teaching of the female teenagers about the use of contraceptives was ignored to such an extent that the perceived blaming of the nurses’ behaviour was cited as a reason for failing to use contraceptives. This became a strong hold which was described by female teenagers as:

‘So I went to one of the clinics. Nurses there are very rude because when you go there they will ask you why you have sex or sleep around while you are still young. What! What!’ (Participant 10)

‘Sometimes nurses can be harsh on you and sometimes they do not give advice. They can tell you why you have sex and stuff instead of helping you out.’ (Participant 14)

### Contraceptive effects on teenagers

Contraceptive effects on teenagers were apparent as side effects coupled with the myths. Side effects were pointed out as being responsible for the gynaecological problems and changes in the body structure. These were verbalised as follows:

‘Uh! I started; I used them for 2 years and then started having pain in the bladder. I ran to hospital and they transferred me to gynaecology. I stopped using them; thinking that maybe it is the clot from the dirty clot. So they transferred me to gynaecology and I told them I do not want to use these conca … those pills and those injections anymore.’ (Participant 6)

‘I used the injection, then stopped it when it stopped my periods. The thing of stopping my periods, I do not like it.’ (Participant 13)

‘There is this thing that; that is said; when you use contraceptives you gain weight or you start eating too much things like that. So they would rather not use them because we are afraid of gaining weight, ja.’ (Participant 11)

Some of the participants described their own and their acquired ideas about contraceptive use in this manner:

‘I was thinking that they will not treat me well. So many people, who use them, say the contraceptives leave dirty blood in your body.’ (Participant 5)

‘I stopped using them because I thought it is a dirty clot.’ (Participant 6)

### Social pressure

The researcher observed that the participants in this study adhered to the controlling directives they obtained from friends and other people of importance. Peer influence and influence by significant others contributed a lot of pressure on the participants’ contraceptive use. The pressure caused the participants to give in to the demands of their peers and significant others, to such an extent that participants imitated each other's behaviour. This observation is supported by one of the participants, who said:

‘I was thinking I would not have sex. So there comes a stage of adolescent. So me and my friends, we imitated one another. It was exciting. We then had sex secretively. When having sex secretively like that, then came another part. I fell pregnant and my friends did not.’ (Participant 5)

The participants believed in their friends and significant others’ approval, or disapproval, to such an extent that it served as justification for the use or non-use of contraceptives:

‘As we are like talking as teenagers some teenagers said even if you use them the sperm will enter inside you so is like there is no use to use them.’ (Participant 12)

‘My grandmother did not want me to use the contraceptives. She thinks that I am going to be all over [*promiscuous*].’ (Participant 7)

‘I am going to use it (contraceptive methods) because the nurse said so.’ (Participant 7)

### Education about contraceptives

The participants verbalised the following perception that contributed to non-use and/or discontinued use of contraceptives: The need for more information and reinforcement of what is known. Some participants felt there is a strong need to involve peers and others in education about contraceptives:

‘I think if the teenager can talk to them because if an elder talks to them they think that, that elder, is she is mad or does not know anything because today's youth think that they know everything and that is not the right thing. We all know that we do not know anything; we are still learning.’ (Participant 13)

Another participant indicated that:

‘Advice must always be given to them. I think mostly doctors and nurses must go to the schools to go and talk to teenagers about this contraceptive thing because most or some of them do not get … do not learn this from their parents. Some parents are scared to talk to their children about having sex at an early age and what could happen to them. So, I think teenagers must be given advice about contraceptives.’ (Participant 14)

## Ethical considerations

University of Pretoria Faculty Ethics Committee (105/2013), the Tshwane District Ethics Committee (20/2013) and the facility manager approved the research. Participants were alerted and informed about what their participation involved, with emphasis on voluntary participation. Thereafter, verbal informed consent and written informed consent were granted by individual participants prior to the interview. Female teenagers under the age of 18 had to discuss their participation with the parents, agree and be allowed participation by their parents through a signed informed consent form. The participants brought the forms to the appointments on the dates set with the researcher. Ethical principles of research were adhered to.

### Measures to ensure trustworthiness

Guba's approach was used in this study to ensure the study rigor (Polit & Beck 2012:584–595). Credibility was obtained through the use of prolonged engagement with the participants and member checking. Member checking was ascertained by reiterating information received from the participants during the interview discussions, at the end of each interview session. On the other hand, prolonged engagement was ascertained through involvement with the participants during data collection, including 30 to 45 minutes of interview on a face-to-face encounter. The researcher submitted raw data to the independent coder to achieve confirmability of this study. Thereafter, rich descriptive data was provided in the research report to allow transferability.

## Discussion

According to the results, in the North-West area of the Tshwane District perceptions of female teenagers contributed to non-use and discontinued use of contraceptives. Most female teenagers in the Tshwane District still experienced unplanned pregnancies. From the 15 female participants interviewed, perceptions on the use of contraceptives, contraceptive effects on teenagers, social pressure and education about contraceptives were described as contributing to the use or non-use of contraceptives. The perceptions on the use of contraceptives were revealed. Although some were positive, the predominant perceptions were negative, showing some ignorance and a tendency to blame others. Syed *et al.* (2012:2–5), Appiah-Agyekum and Kayi (2013:41–42) and Kinaro (2013:7) are in agreement with the findings that contributed to the non-use and the discontinued use of contraceptives.

Of concern were the predominating negative perceptions. Lack of willingness to utilise contraceptives reflected the greatest negative perception described by female teenagers. Despite the knowledge and understanding of the value of contraceptive use female teenagers displayed, there were still utterances such as the use of contraceptives was stressful and too much of a responsibility, which revealed ignorance and lack of commitment. Khanal *et al.* (2011:181) pointed to the same sentiment in a similar study they conducted. Female teenagers seemed to have allowed themselves to be overwhelmed by their perceptions to the point of blaming the nurses for their failure to use contraceptives.

Contraceptive effects on teenagers included perceived side effects and myths shared by female teenagers as they related to one another during the discussions. The researcher observed that perceived contraceptive effects discouraged the majority of female teenagers in the Tshwane District to use or continue with the use of contraceptives. The focus was on *petty* issues such as change in looks than on the unpleasant consequences of non-use of contraceptives. Female teenagers complained of weight gain, change in menstrual patterns, being sick and thinking that contraceptives induce dirty blood or clots. Their perceptions contributed to a lack of initiation of contraceptives by some of the teenagers, which the majority regretted. Evidence in studies conducted by Kanku and Mash (2010:568), Okanlawon *et al.* (2010:20), Wood *et al.* (2009:20) and Hagan and Buxton (2012:176) confirm the findings of this study.

The researcher discovered that the female teenagers subjected themselves to invaluable influence and pressure by peers and significant others. The participants lacked belief in their ability to make decisions regarding contraceptive use, as such conformed to the messages they received from their peers and significant others. The unpleasant outcome, which was unplanned pregnancy, made them become teenage mothers; a mistake they regretted immensely. The participants felt they could have used the contraceptives to delay pregnancy despite the perceptions. Thus, they could have avoided the mistake of falling pregnant at a young age. Forty percent of female teenagers between the ages of 15 and fewer than 20 were reported as being influenced not to use contraceptives (Aryeetey *et al.* 2010:30).

The researcher noted that the majority of female teenagers cited the need for more information should be accessible to their peers and significant other people. Provision of information on available contraception became a strong desire from the discussions held with the participants. Female teenagers believe that a lack of information or discussion with parents about contraception has affected their contraceptive use behaviour (Kanku & Mash 2010:568). Therefore, the researcher is of the opinion that there is a need to rethink education programmes and adapt them to meet the needs of female teenagers to encourage and sustain contraceptive use, prevent accidental pregnancies and minimise the unpleasant consequences they experienced. Attention should be focused on other available methods of contraception as the condom, pill, and injectables were the only methods of contraception used for those who ever used, or discontinued using, them. Side effects should be mentioned, and the actions be taken when experiencing the side effects. The re-education should be the effort of a collaborative partnership amongst female teenagers, nurses, doctors, teachers and community groups to effectively improve contraceptive use.

### Implications of the study

The findings in this study reflected the perceptions of female teenagers in the Tshwane District. The need for joint efforts amongst clinic nurses, managers, lecturers and doctors in educating female teenagers about other available contraceptive methods should be of high priority in order to increase teenagers’ choice of method and to influence change in their perceptions on contraception. The researcher recommends that health education programmes be restructured to intensify education on contraception and contraceptive methods. The purpose of such programmes would be to effectively influence female teenagers’ perceptions about contraception and encourage commitment and informed decision-making regarding their use. The programmes should incorporate community groups, parents and male partners to enhance ownership and effective dissemination of information.

## Limitation of the study

The source of the data was largely dependent on female teenagers within the age of 15 to 19 in a selected clinic. As such, the findings cannot be transferable since the study is not representative of the entire teenage population.

## Recommendation of the study

The study recommends that health education should be structured to meet the needs of teenagers. The study further suggests that research should be conducted which should focus on the perceptions of both female and male teenagers on the use of contraceptives on a larger scale, as this study only investigated female teenagers in a selected clinic.

## Conclusion

The results provided evidence-based information about the perceptions described by the female teenagers in the Tshwane District. The findings revealed that the perceptions of female teenagers on the use of contraceptives were a major reason for female teenagers not using, and discontinuing using, contraceptives. The majority of participants displayed an intention of preparedness to comply with the use of contraceptives to avoid the mistakes of encountering the unpleasant outcomes they were faced with. Thus, the need for more information from knowledgeable persons. The female teenagers’ perceptions can be positively influenced to improve the behaviour regarding the use of available contraceptives.
